# Lesson from COVID-19—Adapting Respiratory Rehabilitation Through Early Multidisciplinary Care: An Opinion Paper from Retrospective Data

**DOI:** 10.3390/jcm14113745

**Published:** 2025-05-27

**Authors:** Federica Bellone, Adriana Tisano, Giulia Leonardi, Daniela Amato, Daniele Borzelli, Giuseppe Santoro, Angelo Alito, Francesca Cucinotta, Simona Portaro

**Affiliations:** 1 Department of Clinical and Experimental Medicine, University of Messina, 98125 Messina, Italy; federica.bellone@unime.it (F.B.); atisano@unime.it (A.T.); 2 Physical Rehabilitation Medicine Department, University Hospital A.O.U. “G. Martino”, 98124 Messina, Italy; giulia.leonardi@polime.it (G.L.); daniela.amato@polime.it (D.A.); simonaportaro@hotmail.it (S.P.); 3 Department of Biomedical, Dental Sciences and Morphological and Functional Images, University of Messina, 98125 Messina, Italy; daniele.borzelli@unime.it (D.B.); giuseppe.santoro@unime.it (G.S.); alitoa@unime.it (A.A.); 4 IRCCS Centro Neurolesi Bonino-Pulejo, 98124 Messina, Italy

**Keywords:** Barthel Index, COVID-19, chronic respiratory diseases, early rehabilitation, functional assessment, multidisciplinary care

## Abstract

The COVID-19 pandemic significantly challenged healthcare systems worldwide, highlighting the critical role of early and multidisciplinary rehabilitation interventions. There remains uncertainty about whether these rehabilitation principles developed in response to COVID-19 can be effectively generalized to chronic respiratory diseases. This opinion paper evaluates retrospective data from early stages of the COVID-19 pandemic to propose a comprehensive early multidisciplinary rehabilitation model, potentially applicable across various chronic respiratory conditions. We retrospectively analyzed data from 62 COVID-19 patients hospitalized at the University Hospital of Messina, Italy, between March and June 2020. The patients underwent structured multidisciplinary rehabilitation sessions initiated upon achieving clinical stability. Functional improvements were assessed weekly using the Barthel Index (BI). Significant correlations were observed between initial BI scores and functional improvements at discharge (*p* < 0.001) and between functional gains and the number of rehabilitation sessions administered (*p* = 0.025). Early physiatric evaluation significantly enhanced functional recovery (*p* = 0.036). Early structured multidisciplinary rehabilitation during acute hospitalization demonstrated clear benefits in COVID-19 patients, indicating potential for broad applicability in chronic respiratory disease management. Systematic implementation could improve clinical outcomes, reduce healthcare resource utilization, and enhance patients’ quality of life.

## 1. Introduction

The emergence of Severe Acute Respiratory Syndrome Coronavirus 2 (SARS-CoV-2) in December 2019 strongly challenged healthcare systems worldwide [1,2,3]. Coronavirus disease 2019 (COVID-19) presented with various clinical manifestations, from mild influenza-like symptoms to severe forms characterized by acute respiratory distress syndrome (ARDS), multisystem organ failure, and extended periods of critical illness [4,5].

While COVID-19 primarily affects respiratory function, extensive evidence suggests it should be regarded as a systemic condition impacting multiple organ systems, including cardiovascular, neurological, hematopoietic, and immune systems [6,7,8].

Rehabilitation strategies developed in response to the COVID-19 crisis may offer valuable insights into the management of respiratory diseases.

Can these strategies serve as a translational model for managing chronic respiratory diseases?

Recent literature underscores that early and multidisciplinary rehabilitation initiated during acute illness, including intensive care unit (ICU) stays, significantly improves long-term patient outcomes, reducing disability and promoting faster functional recovery [6,9]. The exceptional contexts of the COVID-19 pandemic imposed rapid innovation and adaptation, providing a unique opportunity to reconsider established rehabilitation protocols and explore novel multidisciplinary approaches [10,11]. This perspective offers interesting possibilities for the transfer of these findings to chronic respiratory diseases, such as chronic obstructive pulmonary disease (COPD), idiopathic pulmonary fibrosis (IPF), and post-ARDS sequelae of various etiologies.

Furthermore, contemporary studies indicate a clear necessity to optimize healthcare resources by implementing early interventions that improve patient outcomes while concurrently reducing hospital stays, readmission rates, and overall healthcare costs [12,13]. Rehabilitation programs traditionally implemented during later stages of chronic respiratory disease progression have limited impact on reversing significant functional decline [14]. Conversely, early structured interventions may considerably correct patient paths, reduce subsequent healthcare utilization, and enhance quality of life, presenting significant economic benefits for strained healthcare systems [15].

These findings raise the possibility that what we have learned under the pressure of the pandemic could evolve into a new paradigm, offering a potential way forward to better support people living with chronic and disabling respiratory conditions.

Initiating pulmonary rehabilitation programs after hospital discharge or during the stable chronic phase limits their effectiveness in reversing deconditioning and impairs long-term prognosis [16]. Furthermore, many healthcare systems still lack multidisciplinary integration, which is often limited by logistical, economic, or structural issues.

The 2023 Global Initiative for Chronic Obstructive Lung Disease (GOLD) report emphasizes the importance of multidisciplinary and early interventions but provides few practical guidelines for implementing these strategies during acute hospitalizations [17].

Recent studies have shown that early physiotherapy and mobilization during hospitalization may improve outcomes for patients with chronic respiratory disease [18], but randomized evidence remains scarce, and structured protocols applicable to broader populations are lacking. The pandemic presented an unexpected opportunity to advance the implementation of early, intensive, and multidisciplinary rehabilitation approaches, even for critically ill patients. It has been shown that these interventions, which were driven by necessity, are safe, feasible, and potentially impactful [19,20].

Early rehabilitation intervention is crucial for optimizing outcomes for respiratory patients [21]. A recent study found that starting respiratory rehabilitation within four days of admission significantly reduced disease progression and mortality, even among critically ill patients. Multimodal approaches are safe and feasible in acute care, reinforcing the value of early rehabilitation for chronic respiratory diseases [19].

The effective delivery of such interventions depends on the coordinated involvement of a multidisciplinary team. This typically includes the medical team, physiatrists, physiotherapists, respiratory therapists, nurses, and intensive care specialists working closely with psychologists and occupational therapists. The integration of these professionals allows for a comprehensive and patient-centered approach that addresses not only respiratory and functional impairments but also the neurocognitive, psychological, and nutritional sequelae of COVID-19 [20,22]. Shared clinical protocols and continuous interdisciplinary communication are essential to adapt rehabilitation strategies to the evolving clinical course of the patient and to maximize safety and functional recovery while minimizing complications.

This opinion paper aims to retrospectively describe the functional outcomes associated with an early, structured, multidisciplinary rehabilitation program administered to hospitalized patients with acute SARS-CoV-2 infection. A secondary aim is to explore potential associations between rehabilitation variables (i.e., timing, number of sessions, early approach) and improvements in functional status. In addition, this paper aims not only to challenge existing paradigms but also to encourage proactive adoption of tailored rehabilitation strategies with demonstrable cost-effectiveness and enhanced patient-centered outcomes.

## 2. Materials, Methods, and Results

We retrospectively reviewed data from 62 patients admitted to the University Hospital of Messina (Italy) between March and June 2020 with a confirmed COVID-19 diagnosis (Ethical Committee approved the study on 9 November 2021 (protocol number 40-21 10 March 2021)). Eligible patients were identified in intensive care units (ICU, n = 25) and acute care units (internal medicine, pulmonology, infectious diseases departments, n = 37). Demographic and functional data are provided in Table 1. Once patients were clinically stabilized during hospitalization in the ICU or acute care units, the physiatrist assessed their physical and neurological condition, blood exams, ventilation type, oxygen saturation, and the absence of any contraindications to start rehabilitation. According to the literature [23], the exclusion criteria for starting rehabilitation were (1) signs of respiratory distress; (2) the need for respiratory support with a fraction of inspired oxygen (FiO_2_) > 60%; (3) the need for continuous positive airway pressure (CPAP); (4) signs of cardiovascular instability or chest compression; and (5) psychomotor agitation or resistance to treatment. Rehabilitation interventions began upon achieving stable respiratory and hemodynamic parameters, irrespective of nasopharyngeal swab positivity, until hospital discharge.

The patients participated in daily rehabilitation sessions (~40 min), and functional assessments were performed weekly using the Barthel Index (BI).

In the ICU, rehabilitation started as soon as respiratory and hemodynamic stability was achieved. The physiotherapy plan was personalized and guided by the level of consciousness, ventilatory support, and clinical parameters. Core interventions included passive and active assisted mobilization, frequent postural changes (including sitting or semi-orthopedic positions), early verticalization when possible, trunk control exercises, and static standing balance. Respiratory rehabilitation focused on improving deep lung ventilation and secretion clearance. Exercises were performed with rest periods to compensate for fatigue, and continuous monitoring was required to interrupt treatment in the event of fever, oxygen desaturation (SpO_2_ < 90%), dyspnea, or clinical deterioration. If possible and tolerated by the patient, muscle strengthening, stretching, and ambulation exercises helped with gradual recovery.

The patients on acute wards often had significant deconditioning due to immobilization, inflammation, and prolonged oxygen therapy. Physiotherapy focused on restoring functional autonomy and endurance through active mobilization, progressive resistance exercises, coordination tasks, and respiratory muscle training. Out-of-bed sitting, sit-to-stand training, early gait re-education, and ADL retraining were progressively introduced. Trunk control and dynamic balance exercises supported neuromotor recovery, while oxygen saturation and other clinical parameters guided intensity. Volume-based breathing strategies, including deep, slow inspirations with inspiration, supported lung recruitment.

In all settings, interventions were tailored to each patient’s clinical status, with particular attention to respiratory, cardiovascular, musculoskeletal, and cognitive functions.

**Figure 1:**
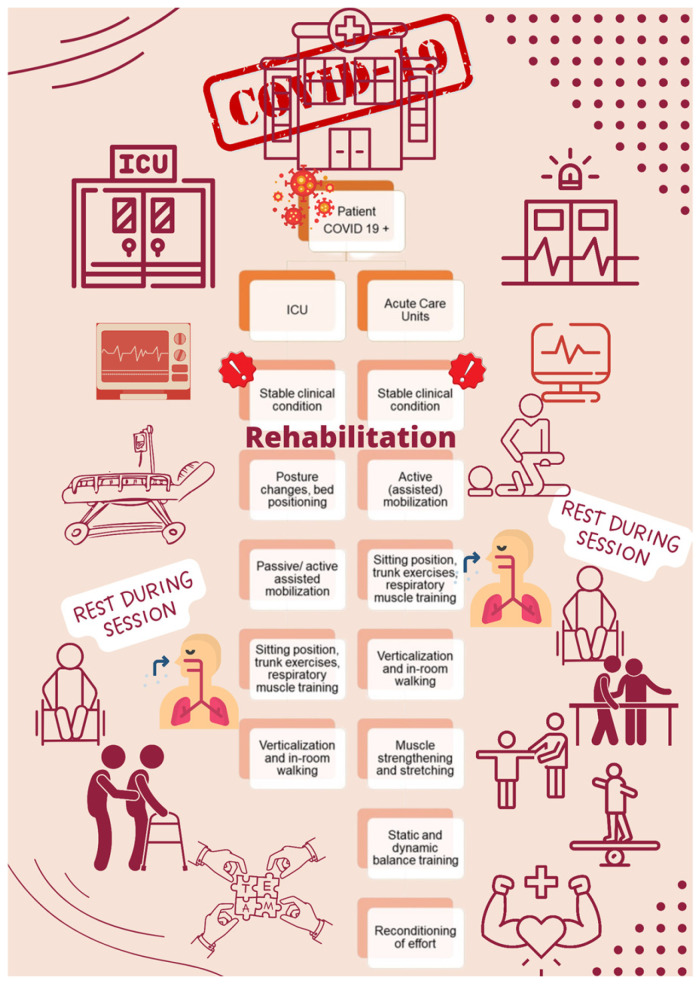
Rehabilitation pathways for patients with COVID-19, based on clinical setting and stability. This flowchart illustrates the different approaches to rehabilitation for hospitalized COVID-19 patients. Once clinical stability has been achieved, rehabilitation is introduced progressively with tailored interventions. The figure highlights the importance of rest during sessions and emphasizes the need for a multidisciplinary team approach to ensure safe, personalized progression.

Respiratory techniques, such as diaphragmatic or pursed-lip breathing, were avoided in the acute phase. The treatment plan and exercises were tailored to each patient’s clinical condition, comorbidities, and tolerance levels to ensure safety and maximize functional recovery. Figure 1 shows a schematic representation of the different rehabilitation pathways.

A linear model (Matlab^®^ v2023b function fitlm) tested the effects of the following factors on the BI at discharge: (i) BI at the first physiatric evaluation, (ii) days of hospitalization before physiatric examination, (iii) number of physiotherapy treatments administrated, (iv) age, and (v) sex. Our data showed that the days before physiatric examination affected the BI at discharge but not linearly. Therefore, the effect of the days before physiatric examination was modelled as a dummy variable, whose value was equal to 1 if the patient was examined in less than d days, where d ranged from 5 to 43, and 0 otherwise. A different linear model was built with each d, while the other tested effects were (a) BI at the first physiatric evaluation, (b) number of physiotherapy treatments administrated, (c) age, and (d) sex.

**Figure 2:**
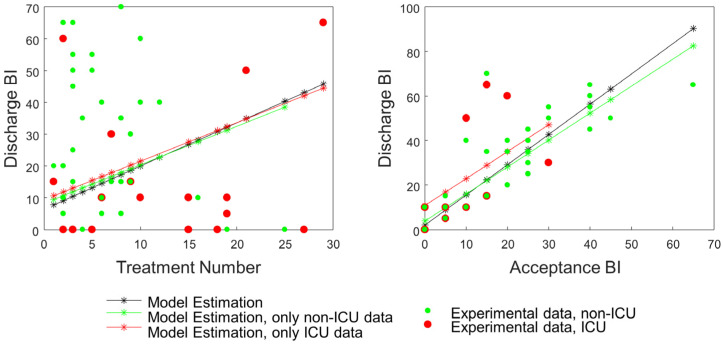
Experimental data and linear model of discharge BI. Experimental data collected from patients who underwent ICU treatment are shown as red dots; data from patients who did not are shown as green dots. The black line with asterisks represents the linear model fitted to data from all patients. The red and green lines with asterisks represent the linear models fitted to data from patients who underwent ICU treatment and those who did not, respectively. (**Left panel**): effect of treatment number on discharge BI. (**Right panel**): effect of admission BI on discharge BI.

The linear model showed that, as expected, the discharge BI was significantly influenced by the BI measured on admission (*p* < 0.001, Figure 2, left panel, black line) and by the number of treatments given to the patient (*p* = 0.025, Figure 2, right panel, black line), with each additional treatment resulting in an average improvement of 1.55 in the discharge BI. In contrast, the discharge BI was not affected by age (*p* = 0.481), sex (*p* = 0.319), or days prior to the medical examination (*p* = 0.324).

A further stratification was performed by dividing the patients who underwent ICU treatment from those who did not. Accordingly, linear models were built on two separate datasets: one comprising the patients who underwent ICU treatment and the other comprising those who did not. The effects of the following factors on the BI at discharge were tested: (i) BI at the first physiatric evaluation, (ii) days of hospitalization before physiatric examination, (iii) number of physiotherapy treatments administrated, (iv) age, and (v) sex.

In line with the model built on the full dataset, discharge BI was significantly influenced by BI at admission in both groups: the patients who underwent ICU treatment (*p* = 0.011; Figure 2, left panel, red line) and those who did not (*p* < 0.001; Figure 2, left panel, green line). Similarly, discharge BI was not affected by age (*p* = 0.405 and *p* = 0.993, respectively, for ICU and non-ICU groups), sex (*p* = 0.728 and *p* = 0.285), or days before the medical examination (*p* = 0.167 and *p* = 0.824). Interestingly, the number of physiotherapy treatments significantly affected discharge BI only in the patients who underwent ICU treatment (*p* = 0.037; Figure 2, right panel, red line) but not in those who did not (*p* = 0.485; Figure 2, right panel, green line).

However, linear models testing the effect of days to examination as a dummy variable identified a significant effect on discharge BI, with an average 8.8 higher value if the examination was performed within 30 days (*p* = 0.036). Examinations performed within fewer days had no effect on discharge BI (see Table 1). The effect of the other parameters did not change with the different threshold in days to examination, and while the number of treatments (*p* < 0.037 in all conditions) and the admission BI (*p* < 0.001 in all conditions) showed a significant effect on the discharge BI, age (*p* > 0.302 in all conditions) and sex (*p* > 0.218 in all conditions) did not.

## 3. Discussion

The retrospective data strongly support the effectiveness of an early, structured, multidisciplinary rehabilitation protocol, inviting broader consideration of how acute care pathways for chronic respiratory diseases could be restructured beyond the immediate context of the pandemic. The significant improvements in functional outcomes highlight the necessity and effectiveness of early initiation of rehabilitation, even in intensive care settings. Recent literature reinforces that initiating rehabilitation early in ICU settings significantly reduces morbidity, decreases the length of hospital stay, and improves overall functional recovery [24,25]. Continuous rehabilitation through subsequent inpatient settings further consolidates these functional gains, highlighting the importance of seamless rehabilitation care transitions [26].

BI is confirmed as a reliable prognostic tool for both mortality and functional outcomes, particularly in older patients with COVID-19. Our findings are consistent with data from the Italian RePoSI Registry, in which, in a cohort of 4714 elderly hospitalized patients, alongside glycemia level ≥ 250 mg/dL, Cumulative Illness rating Scale (CIRS-SI), and male sex, a BI ≤ 40 was a stronger predictor of in-hospital mortality for older patients admitted in general wards [27].

Early physiatric evaluation is critical for the timely and targeted initiation of rehabilitation in patients with acute respiratory failure. It allows for early identification of functional limitations and guides the integration of rehabilitation into the acute care pathway, helping to prevent complications such as deconditioning and prolonged immobility and ensuring continuity of care from admission to post-acute recovery [28].

BI assessment enables the identification of individuals at higher risk of disability and poor prognosis, while its longitudinal use provides valuable insight into rehabilitation response and functional progression. The utility of the BI extends beyond mortality prediction, offering a valuable means of evaluating functional outcomes such as post-intensive care syndrome (PICS). Its routine application in clinical practice could facilitate more precise rehabilitation planning and contribute to a more efficient allocation of healthcare resources [29,30].

The principles of early mobilization and structured respiratory physiotherapy reinforce our rehabilitation protocol. Early mobilization addresses the severe muscular and cardiovascular deconditioning prevalent in critically ill respiratory patients, suggesting similar benefits could extend effectively to chronic conditions like COPD and IPF [31,32].

The application of respiratory physiotherapy, including postural drainage and respiratory muscle training, is well supported by current literature as essential in managing chronic respiratory diseases, reducing complications, and improving pulmonary function [33]. Implementing early multidisciplinary rehabilitation for chronic respiratory diseases would require redefining the timing, roles, and resource allocation across clinical settings. This would entail structured training programs, early physiatric involvement, and protocols for individualized progression. Most importantly, it would demand a cultural shift in how we approach chronic disability in respiratory patients, moving from a reactive to an anticipatory care model.

The multidisciplinary approach involving physiotherapists, respiratory therapists, psychologists, and physicians significantly contributed to comprehensive, patient-tailored rehabilitation [34,35], improving patient adherence, optimizing healthcare resources, and improving outcomes across a range of respiratory diseases through the speculative but practical integration of such multidisciplinary care across the board.

The COVID-19 pandemic has served as a strong catalyst for the development of early pulmonary rehabilitation strategies for hospitalized patients with acute respiratory failure. Recent studies consistently highlight multidisciplinary interventions as essential to improving chronic respiratory disease management outcomes. A multidisciplinary approach significantly enhances the quality of life and health outcomes for individuals with COPD [36]. The 2023 Global Initiative for Chronic Obstructive Lung Disease (GOLD) report emphasizes integrated multidisciplinary care as necessary for effective COPD management [17].

Based on the extensive clinical data collected during the pandemic, a structured model of pulmonary rehabilitation has emerged that has demonstrated significant benefits in improving functional outcomes, reducing disease progression, and improving overall quality of care. Given the similarities in pathophysiology and rehabilitation needs, this model may be applicable to other respiratory diseases, such as acute exacerbation of COPD, ARDS, or severe pneumonia.

This protocol can be broadly divided into three related phases. The initial stabilization phase focuses on ventilatory efficiency, and the prevention of complications related to immobility. Semi-orthopneic or sitting positions are recommended to improve the ventilation/perfusion ratio and pulmonary mechanics. In selected patients with refractory hypoxemia, prone positioning for at least four hours daily may further improve oxygenation, although careful monitoring of hemodynamic and cardiac status is essential during this intervention.

The pulmonary optimization phase focuses on airway clearance and lung recruitment strategies. Airway clearance therapy (ACT), including vibratory positive expiratory pressure and high-frequency chest wall oscillation, is indicated in the presence of rough crackles or high sputum burden, especially in the presence of bacterial coinfection. Respiratory resistance training using incentive spirometry is particularly beneficial in cases with radiological evidence of atelectasis or consolidation. Studies have shown that the inclusion of at least two different pulmonary rehabilitation interventions is associated with greater improvements in respiratory parameters and clinical outcomes [37].

Once respiratory and hemodynamic parameters have stabilized, the patient enters the mobilization and functional recovery phase, where early physical rehabilitation becomes central. Gradual progression from bed-based exercises to sitting, standing, and assisted ambulation forms the basis of this phase. Progressive resistance training, trunk control, and gait retraining are implemented with continuous clinical monitoring. Patients are also encouraged to perform simple activities of daily living, such as sitting up for meals and walking short distances, to increase autonomy and prevent ICU-acquired weakness. Patient safety must guide all phases of rehabilitation. Continuous monitoring of vital signs, especially oxygen saturation, heart rate, and blood pressure, is essential. In patients with cardiovascular comorbidities, baseline cardiac assessment is recommended to minimize the risk of adverse events during exercise. Any clinical deterioration should prompt immediate reassessment or cessation of the intervention.

Functional outcomes should be monitored using validated tools, while improvements in respiratory status can be assessed by changes in the PaO_2_/FiO_2_ ratio.

This structured model, developed during the COVID-19 pandemic, can provide a safe, flexible, and evidence-based framework for pulmonary rehabilitation. When applied within a multidisciplinary team, it can be effectively adapted to other acute respiratory conditions to enhance recovery, reduce disability, and improve patient outcomes.

The authors acknowledge that the retrospective nature of this study, the limited sample size, and the absence of a concurrent control group limit the ability to draw causal relationships. It is important to note that this evidence includes results from retrospective cohort studies, which have inherent limitations, such as potential selection bias and possibly unmeasured confounders. The findings align with existing literature supporting early multidisciplinary rehabilitation, but unmeasured confounders may have influenced the outcomes. These factors highlight the need for caution when interpreting the results as evidence of efficacy. The pandemic experience highlights significant challenges, including logistical constraints and limited resources, emphasizing the need for flexible and adaptable rehabilitation strategies broadly applicable across healthcare systems. Future research should focus on prospective randomized controlled trials on a large scale to validate the proposed rehabilitation model and to improve generalizability across various populations, assessing both clinical effectiveness and cost–benefit sustainability.

## 4. Conclusions

While the findings of this retrospective analysis highlight the potential benefits of early, structured, multidisciplinary rehabilitation in acute respiratory settings, they should consider the limitations. Nevertheless, the observed functional improvements and the feasibility of early intervention suggest that this model should be explored further in broader clinical contexts.

Integrating early rehabilitation into standard respiratory care pathways holds significant translational potential. However, generalizing this approach requires caution due to the heterogeneity of conditions such as COPD, IPF, and post-ARDS syndromes as well as variability in individual patient profiles. Meanwhile, careful implementation in well-resourced settings, with the right training and monitoring, could be a practical way to improve respiratory care.

## Figures and Tables

**Table 1:** Demographic features and functional evaluation of patients.

	ICU	Acute Care Units	Total
Number of patients	25	37	62
Age (y)	65.84 ± 11.55	72.19 ± 12.53	70.50 ± 12.31
Sex	14 M/11 F	10 M/27 F	24 M/38 F
LOS	49.32 ± 29.67	34.13 ± 13.87	40.26 ± 22.90
Tracheostomy	12 (48%)	2 (5.41%)	12 (48%)
Number of treatments	10.92 ± 7.91	6.70 ± 5.22	8.40 ± 6.77
Dead	3 (12%)	2 (5.41%)	5 (8.06%)
Comorbidities (%)			
Arterial hypertension	15 (60%)	28 (75.68%)	43 (69.35%)
Diabetes mellitus	9 (36%)	14 (37.84%)	23 (37.10%)
Dyslipidemia	3 (12%)	8 (21.62%)	11 (17.74%)
Obesity	4 (16%)	8 (21.62%)	12 (19.35%)
Neuromuscular diseases	1 (4%)	5 (13.51%)	6 (9.68%)
Functional assessment			
Days before first physiatric evaluation	23 ± 14	19 ± 12	21 ± 13
BI on admission	8 ± 8	19 ± 15	15.28 ± 14.32
BI at discharge	16 ± 15	29 ± 21	24.17 ± 21.84

BI: Barthel Index; ICU: intensive care unit; LOS: length of stay.

## Data Availability

The data presented in this study are available within this article.

## Abbreviations

The following abbreviations are used in this manuscript:

SARS-CoV-2	Severe Acute Respiratory Syndrome Coronavirus 2
COVID-19	Coronavirus disease 2019
ARDS	Acute respiratory distress syndrome
ICU	Intensive care unit
COPD	Chronic obstructive pulmonary disease
IPF	Idiopathic pulmonary fibrosis
PICS	Post-intensive care syndrome
GOLD	Global Initiative for Chronic Obstructive Lung Disease
